# Improved ability of biological and previous caries multimarkers to predict caries disease as revealed by multivariate PLS modelling

**DOI:** 10.1186/1472-6831-9-28

**Published:** 2009-11-03

**Authors:** Åke Nordlund, Ingegerd Johansson, Carina Källestål, Thorild Ericson, Michael Sjöström, Nicklas Strömberg

**Affiliations:** 1 Department of Odontology/Cariology, Umeå University, 901 87 Umeå, Sweden; 2 Department of Women's and Children's Health/International Maternal and Child Health, Uppsala University, 751 85 Uppsala, Sweden; 3 Department of Chemistry, Umeå University, 901 87 Umeå, Sweden

## Abstract

**Background:**

Dental caries is a chronic disease with plaque bacteria, diet and saliva modifying disease activity. Here we have used the PLS method to evaluate a multiplicity of such biological variables (n = 88) for ability to predict caries in a cross-sectional (baseline caries) and prospective (2-year caries development) setting.

**Methods:**

Multivariate PLS modelling was used to associate the many biological variables with caries recorded in thirty 14-year-old children by measuring the numbers of incipient and manifest caries lesions at all surfaces.

**Results:**

A wide but shallow gliding scale of one fifth caries promoting or protecting, and four fifths non-influential, variables occurred. The influential markers behaved in the order of plaque bacteria > diet > saliva, with previously known plaque bacteria/diet markers and a set of new protective diet markers. A differential variable patterning appeared for new versus progressing lesions. The influential biological multimarkers (n = 18) predicted baseline caries better (ROC area 0.96) than five markers (0.92) and a single lactobacilli marker (0.7) with sensitivity/specificity of 1.87, 1.78 and 1.13 at 1/3 of the subjects diagnosed sick, respectively. Moreover, biological multimarkers (n = 18) explained 2-year caries increment slightly better than reported before but predicted it poorly (ROC area 0.76). By contrast, multimarkers based on previous caries predicted alone (ROC area 0.88), or together with biological multimarkers (0.94), increment well with a sensitivity/specificity of 1.74 at 1/3 of the subjects diagnosed sick.

**Conclusion:**

Multimarkers behave better than single-to-five markers but future multimarker strategies will require systematic searches for improved saliva and plaque bacteria markers.

## Background

Dental caries is a chronic disease [1]. Many western countries show a skewed caries distribution with many healthy and 15-20% diseased subjects [2]. Moreover, traditional regimens for risk assessment and prevention are inefficient for controlling the diseased group [2,3]. Thus, refined etiological and prediction models for caries are needed.

Both lifestyle and genetic factors modify caries activity [1,4]. Accordingly, plaque acidification from frequent sugar intake trigger disease development more rapidly in susceptible than resistant subjects by selecting for cariogenic mutans streptococci and lactobacilli and by dissolving the enamel [5,6]. Individual polymorphisms affect the saliva innate defences, e.g. adhesion of *S. mutans*, and specify individual susceptibility [7-9]. It remains, however, to establish to which degree caries is predictable and how various biomarker strategies should be applied to better explain and predict caries.

A wide variety of quantitative plaque, diet and saliva factors (e.g. mutans streptococci, lactobacilli, sugar intake, buffer effect and pH) have been evaluated, and clinically applied, as risk factors or predictors of future caries [reviewed in [10-12]]. Some studies have argued for a substantial predictive ability of plaque, diet and saliva factors [13], particularly in young children and elderly [14-16]. By contrast, extensive prediction studies in adolescents have generally shown *i*) biomarkers to add only marginal information to the ability of clinical markers (*e.g*. previous caries and clinician's "estimation") to explain 33% or less of the individual variation in caries development, *ii*) a predictive ability in order of previous caries >> bacteria > diet and saliva and *iii*) a sensitivity/specificity around 0.74/0.74 or less for single-to-several marker models [10-12,17-19]. Single-to-several marker models have at best shown a sensitivity/specificity of 0.87/0.83 in infants [16].

Both cross-sectional and prospective studies, where factors are measured at baseline and compared to future caries, have been used to explore biomarkers or predictors for caries [reviewed in [10-12]]. Prospective prediction studies - the golden standard in risk evaluation - are hampered by several factors. First, today caries shows a low prevalence and develops slowly. Refined caries indices recording numbers of incipient and manifest caries have accordingly been suggested but not yet evaluated [20]. Second, traditional regression techniques require a high subject-to-variable ratio (so-called "long and lean" data structures), and most prediction studies have therefore been restricted to a limited set of well-established clinical or traditional factors. Consequently, information on the predictive ability of biological multimarkers is lacking.

Partial least squares projections to latent structures (PLS) are optimally designed to correlate multiple and co-varying descriptor X and response Y variable matrices [21,22]. PLS has been used extensively in quantitative structure activity relationships QSARs [21], in metabonomics, proteomics and genomics [22] as well as applied to medical diseases [8,9,23]. It can handle X variables that by far exceed the number of subjects studied (so-called "short and fat" data structures) and gives explanatory (R^2^) and via cross-validation predictive (Q^2^) values for the y variables.

The purpose of the present study was to test PLS modelling for ability to generate predictive models based on multiple biological and previous caries markers (so-called multimarkers) in a cross-sectional (baseline caries) and prospective (2-year caries development) setting and to screen and rank the multiplicity of individual quantitative plaque, diet and saliva variables used (n = 88) for caries promoting or protecting properties.

## Methods

### Study design and cohort

**Figure 1:**
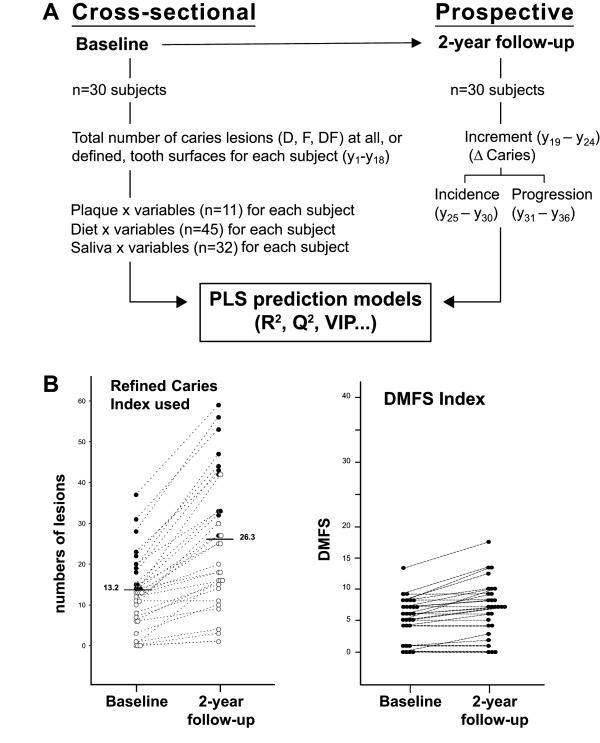
**A. Study design**. A multiplicity of traditional plaque, diet and saliva (n = 88) and caries variables (y_1_-y_36_) were recorded to generate PLS prediction models in a cross-sectional and prospective setting. **B**. **Scoring of caries**. Baseline and 2-year follow-up caries scores for the thirty subjects when using the refined index recording numbers of incipient and manifest lesions at all (or defined) surfaces or the traditional DMFS index.

Aiming at studies on the PLS method as a tool to evaluate and screen biological factors as markers or predictors for caries, we made use of an available cohort material from 1985-87 with many recorded biological variables. Plaque, diet and saliva variables and caries were accordingly recorded at baseline 1985 and at a 2-year follow-up in thirty14-year-old adolescents (mean age 13.9 years, SD 0.4, range 1.5 years) (Fig. 1). The study cohort (17 boys and 13 girls) was randomly selected from the 128 students in a 7^th ^grade of junior high school of a suburban area (Holmsund) outside Umeå, Northern Sweden, with caries levels and life style patterns generally present at that time. All children were reported as healthy and neither medicated nor used tobacco. They were called to the dental health service each year and appropriately treated during and after the study. No dropouts occurred. The study was approved by the Ethics Committee for Human Experiments at Umeå University, Sweden, and informed consent was given by the adolescents and their parents.

### Plaque variables (n = 11)

Biological variables related to plaque (mutans streptococci, lactobacilli, plaque amount, rate, phosphate, fluoride) were measured (n = 8) or derived (n = 3).

The prevalence (% positive surfaces) of mutans streptococci (ms) and lactobacilli (lbc) in interdental plaque was measured as previously described [24]. Briefly, after insertion of toothpicks into the interdental sites distal to the canines in the upper and lower jaws, a replica was made on MSB and Rogosa agar plates and cultured at 37°C for two days in air supplemented with 5% carbon dioxide. Colonies of mutans streptococci and lactobacilli were identified visually and verified by biochemical tests whenever in doubt. The number of interdental sites carrying each species (or the derived combinations lbc^+^/ms^+^, lbc^+^/ms^- ^or lbc^-^/ms^+^, n = 3) was expressed in percent of the total number of available sites.

The numbers of colony forming units (CFU) of mutans streptococci and lactobacilli per ml whole saliva were determined by growth of serial dilutions of saliva on mitis-salivarius-bacitracin (MSB) [25] and Rogosa [26] agar plates, respectively. The plates were incubated at 37°C for two days and counted.

Plaque amount [% surfaces stained positive for plaque, [27]] and rate of formation [28] were measured using different indices before and after tooth cleaning, respectively. Briefly, the rate was estimated by staining of selected teeth (buccal surfaces of upper right canines, premolars or 1^st ^molars) with 1% basic fuchsin after 19-hours absence of oral hygiene following professional tooth cleaning. The rate was scored as rapid if plaque was detected on any surface and as slow if not.

Phosphate and fluoride in plaque fluid were measured. After 4 days without oral hygiene, plaque was sampled from available buccal and lingual surfaces. After addition of buffer and centrifugation (14,000 *g *× 1 h) of the plaque samples at room temperature, the amounts of phosphate (μmol/g wet weight) [29] and ionized fluoride (μg/g wet weight) (Orion^® ^Fluoride Electrode, Orion Research Inc.) in the supernatant were determined.

### Diet variables (n = 45)

The diet variables were estimated from a four day food diary. The diary, starting on a Sunday, was kept by each child with parental support and followed-up on by a trained dietician. The average daily intake of energy (kcal/day) and 44 nutrients (μg, mg or g/day), adjusted for losses due to food preparation, was calculated using the data base from the National Food Administration in Sweden and the software MATs (Rudans Lättdata, Västerås, Sweden). The nutrients are given in the tables or below; disaccharides, total fat, cholesterol, mono unsaturated and saturated fat, myristic acid (14:0), palmitic acid (16:0), stearic acid (18:0), arachidic acid (20:0) palmitoleic acid (16:1), oleic acid (18:1), linolenic acid (18:3), eicosatetraenoic acid (20:4), eicosapentaenoic acid (20:5), arachidonic acid (20:6), docosapentaenoic acid (22:5), docasahexaenoic acid (22:6), β-caroten, vitamin D, niacin, niacin equivalents, thiamine, vitamin C, vitamin B_6_, vitamin B_12_, manganese, selenium and water.

### Saliva variables (n = 32)

Biological variables related to stimulated or resting parotid saliva (agglutinin, S-IgA, lysozyme, peroxidase, thiocyanate, phosphate, calcium and flow rate, n = 16) or to whole saliva (flow rate, pH, buffering, chewing rate, n = 4) were measured or derived (n = 12).

Whole and parotid saliva were collected into ice-chilled test tubes between 1 and 4 pm without drinking or eating in the preceding hour. Whole saliva was stimulated by chewing on paraffin (1 g); the first ml was discarded and 3 ml collected. Both resting parotid saliva, and stimulated by an acidic lozenge (SST™, Salix Pharma, Stockholm, Sweden), were collected using Lashley cups; the first 0.1 ml was discarded and 1.5 ml collected of each type of saliva. Flow rate (ml/min) was calculated. Chewing frequency was recorded (circles/min). The pH and buffer capacity in whole saliva were determined within 30 minutes using a digital pH meter and standard methods (Beckman Instruments, Fullerton, CA).

The resting and stimulated parotid saliva samples were measured for amount/ml of calcium (atomic absorption spectroscopy, Varian Techtron AA6, Varian Associates, Instruments Group, Palo Alto, CA), phosphate [29], thiocyanate (SCN^-^) [30], secretory immunoglobulin A (Immunofluor Technique, Bio-Rad Laboratories, Richmond, CA), lysozyme (Lysozyme Test Kit, Kallestad, Chaska, MN) or activity/ml of peroxidase [31] using standard methods. The two saliva types were also measured for aggregation of *S. mutans *serotype *c *and m-values (activity/ml) were derived as described [32].

The secretion rate/total output of S-IgA, lysozyme, peroxidase, thiocyanate, phosphate and calcium was derived for resting and stimulated parotid saliva (n = 12) by multiplying the amount/ml (or activity/ml) with flow rate (ml/min).

### Caries recordings and outcome measures

Numbers of incipient and manifest caries lesions at all surfaces in the permanent dentition were recorded for each subject at baseline and after 2 years by one examiner (ÅN) using standardized and defined criteria (Additional file 1). Traditional DMFS values were also calculated. The recordings utilized professionally cleaned, air dried, teeth and new dental mirrors and explorers. Bilateral bitewing radiographs were taken and processed manually at the Department of Oral and Maxillofacial Radiology, Umeå University. A total of 264 decayed and 133 filled lesions were recorded at baseline and 299 new and 73 progressing lesions at the 2-year follow-up.

For each subject, a set of 18 baseline caries outcome y variables (y_1_-y_18_) were generated from the total number of decayed (incipient and manifest), decayed/filled or filled lesions at all surfaces (occlusal, buccal, lingual and proximal) or at smooth (buccal, lingual and proximal), proximal, buccal, lingual and occlusal surfaces. A further set of six y variables each were generated from the total number of decayed lesions at the aforementioned surfaces for new (incidence y_19-24_) or progressing (progression y_25-30_) lesions and for their combined numbers (increment, y_31-36_).

### Projections to latent structures by means of partial least squares (PLS)

The multivariate PLS method relates two data matrices, X and Y, to each other by a linear multivariate prediction model. The X matrix consists of predictor × variables and the Y matrix of the corresponding caries outcome y variables for each subject. PLS handles few or many, noisy, collinear, and incomplete variables in both X and Y [21,22]. The precision of the PLS model increases with the increasing number of relevant x and y variables. It generates a number of model (*e.g*. R^2^, Q^2^) and variable (*e.g*. VIP-values, regression coefficients) characteristics and graphic plots. The R^2 ^value gives the capacity of the X-matrix to explain (R^2^) the variance in the Y matrix (and in individual y variables) while Q^2^, generated by cross-validation of blocks of the data, gives its ability to predict (Q^2^) the variance or variation in Y (or in individual y variables). Optimally, the R^2 ^and Q^2 ^values should be as high and close to each other as possible; a Q^2^-value < 0.1 (< 10% predicted variation) reflects a weak model and the combined R^2 ^and Q^2 ^values gives the performance of the PLS model (Table 1). The VIP-value (Variables of Importance in the Projection) summarizes the relative importance of each variable to the X and Y association structure, and variables with a VIP > 1.0 or >1.5 reflects influential or highly influential variables, respectively. Together with regression coefficients for the direction (positive or negative) of each variable association, the VIP value summarizes the behaviour of each variable in the model (Table 2). VIP-values are also used for variable selection for the generation of secondary models.

**Table 1:** Ability of biological and previous caries variables to predict caries

		**Caries Y matrices**^a^							
		
		Baseline		Increment		Incidence		Progression	
					
**X matrices**^a^	**[n variables]**^b^	**R**^2^	**Q**^2^	**R**^2^	**Q**^2^	**R**^2^	**Q**^2^	**R**^2^	**Q**^2^
Primary models (all VIPs)									
Plaque, diet, saliva	[88]	0.597	0.260^M1^	0.619	0.084	0.534	0.056	0.632	0.034
Plaque, diet, saliva + previous caries	[88 + 18]			0.567	0.211^M2^	0.406	0.164^M3^	0.365	0.162^M4^
									
Secondary models (VIPs > 1.0)									
Plaque markers	[6,4,6,4]	0.353	0.289	0.218	0.142	0.181	0.097	0.286	0.226
Diet markers	[7,9,9,0]	0.155	0.113	0.303	0.153	0.170	0.083	nt^c^	nt^c^
Saliva markers	[5,5,7,2]	0.129	0.034	ns^d^	ns^d^	ns^d^	ns^d^	nt^c^	nt
									
Multimarkers (n = 6-39)									
Plaque, diet, saliva	[18,18,22,6]	0.528	0.404	0.377	0.224	0.351	0.204	0.311	0.212^e^
Previous caries	[ ,17,17,16]			0.382	0.323	0.287	0.247	0.494	0.421
Plaque, diet, saliva + previous caries	[ ,35,39,22]			0.662	0.354	0.418	0.318	0.511	0.386
									
Five marker panel^f^	[5]	0.430	0.275						
Single lactobacilli marker^g^	[1]	0.340	0.270						

^a ^The primary models harbor all variables. The secondary models harbor influential (VIP > 1.0) markers from the corresponding primary models M1 to M4. All models had ≤ 3 significant components except for the model with a single saliva lactobacilli marker. The R^2 ^(explanatory) and Q^2 ^(predictive) values gives the performance of each PLS model (see Methods).

^b ^Numbers of variables or markers in each separate PLS model.

^c ^nt = not tested

^d ^ns = no significant component.

^e ^No available diet variable with VIP > 1.0.

^f ^Saliva lactobacilli, plaque mutans streptococci, diet retinol and zinc, saliva calcium with high VIP-values.

^g ^Saliva lactobacilli.

**Table 2:** Association of selected variables or markers with caries.

			PLS associations^a^							
			
x variables or markers		**units**^b^	Baseline		Increment		Incidence		Progression	
						
			VIP	coeff	VIP	coeff	VIP	coeff	VIP	coeff
**PREVIOUS CARIES**^c^										
Decayed, smooth surfaces		numbers/subj			**2.049**	+	**2.221**	+	**2.752**	+
Decayed and Filled, all surfaces		-"-			**1.785**	+	**1.946**	+	**2.204**	+
Decayed, all surfaces		-"-			**1.979**	+	**2.187**	+	**2.427**	+
Decayed, proximal surfaces		-"-			**1.758**	+	**1.841**	+	**2.652**	+
Filled, all surfaces		-"-			**1.478**	+	**1.648**	+	**1.705**	+
Filled, occlusal surfaces		-"-			**1.239**	+	**1.383**	+	**1.576**	+
										
**PLAQUE**^d^										
Lactobacilli	saliva	CFU/ml	**2.494**	+	**1.177**	+	**1.078**	+	**1.982**	+
	plaque	%	**2.244**	+	**1.601**	+	**1.707**	+	**2.041**	+
Mutans streptococci	saliva	CFU/ml	**1.066**	+	0.91	+	0.94	+	0.47	+
	plaque	%	**1.142**	+	**1.104**	+	**1.214**	+	**1.266**	+
Plaque amount		%	0.63	+	0.94	+	**1.020**	+	0.76	+
Plaque formation rate		slow/rapid	0.47		0.98	-	0.46	-	0.72	-
Plaque fluoride		ug/g wet plaque	**1.117**	-	0.75	-	0.88	-	0.58	-
										
**DIET**^e^										
Zinc		mg/day	**1.016**	-	**1.063**	-	**1.194**	-	0.50	-
Lauric acid (12:0)		g/day	**1.115**	-	**1.162**	-	**1.320**	-	0.87	-
Calcium		mg/day	0.92	-	**1.317**	-	**1.750**	-	0.004	-
Riboflavin		mg/day	1.02		**1.047**	-	**1.314**	-	0.27	-
Phosphate		mg/day	1.05		**1.090**	-	**1.320**	-	0.16	-
Protein		g/day	1.05		**1.014**	-	**1.202**	-	0.19	-
Retinol equivalents (vit A)		mg/day	**1.307**	-	0.96	-	**1.102**	-	0.74	-
Buturic acid (4:0)		g/day	0.80	-	0.93	-	**1.185**	-	0.56	-
Linoleic acid (18:2)		g/day	**1.122**	-	0.92		0.60	-	0.29	-
Folate		ug/day	**1.001**	-	0.99	-	**1.130**	-	0.80	-
Vitamin E		mg/day	**1.033**	-	0.95	-	0.89	-	0.72	-
Iron		mg/day	0.97		**1.038**	+	0.43	+	0.08	-
Carbohydrates (total)		g/day	1.10		**1.253**	+	0.80	+	0.28	+
Fiber		g/day	0.77	-	**1.193**	+	0.58	+	0.22	-
Energy		kcal/day	1.19		0.98	+	0.08	+	0.07	-
Monosaccharides		g/day	0.61	-	0.48	+	0.06	-	0.56	-
Sucrose		g/day	1.08		0.87	+	0.43	+	0.37	+
										
**SALIVA**^f^										
*Parotid, stimulated*										
Calcium		mmol/ml	**1.188**	-	0.63	-	0.51	-	**1.111**	-
Phosphate		mmol/ml	0.97		**1.251**	-	**1.260**	-	0.25	+
Aggregation by *S. mutans*		m-value	0.85	+	0.65	+	0.80	+	0.41	+
S-IgA		ug/ml	1.18		0.30	-	0.03	-	0.47	+
Lysozyme		ug/ml	0.51	+	0.75	-	0.08	-	0.80	-
Peroxidase		activity/ml	0.40	+	0.40	+	0.54	+	0.54	-
Thiocyanate		mmol/ml	0.55		0.19	-	0.14	-	0.43	+
Flow rate		ml/min	1.03		**1.020**	+	**1.155**	+	0.35	-
*Whole saliva, stimulated*										
Flow rate		ml/min	1.00		0.36	-	0.22	-	**1.081**	-
pH		pH	0.50		0.39	-	0.34	-	0.20	-
Buffer capacity		final pH	0.61		0.77	-	0.88	-	0.40	-

^ab ^Primary PLS models M1 to M4. VIP values > 1.0 marks influential variables (see Methods). Consistent positive (+) or negative (-) association for the regression coefficients for each y variable; otherwise blank.

^b ^Variable units and units of measures used for derived variables.

^c ^Some of the previous caries x variables (n = 18) derived from the y1-y18 baseline caries variables (see Methods).

^d ^The plaque variables except for phosphate and plaque % of lbc^+^/ms^+^, lbc^+^/ms^- ^or lbc^-^/ms^+ ^(see Methods).

^e ^Some of the 45 diet variables (see Methods).

^f ^The whole saliva variables except for chewing rate. The parotid saliva variables except for the same factors in resting parotid saliva and derived secretion rates for some components in both salivas (see Methods).

### PLS models

A number of primary and secondary PLS models (Table 1) were generated by the Simca software (Simca 11, Umetrics AB, Umeå). The primary PLS models contained all individual x and y variables, while the secondary models contained all or defined blocks or sets of influential (VIP > 1.0) x variables and excluded the occlusal caries y variables in the Y matrices due to individual Q^2^-values below 0.1 (10%). The baseline y_1_-y_18 _variables were also used as previous caries markers or predictors in the prospective setting. The variables were scaled, mean-centered and, when appropriate, logarithmically transformed before subjected to PLS modelling. Moreover, prior to PLS modelling, separate principal component analyses were done on the X and Y blocks to check for homogeneity, *i.e*. lack of strong clustering of the studied subjects.

### ROC curves and sensitivity and specificity calculations

Receiver operating characteristic (ROC) curves which plot sensitivity versus 1-specificity for each possible cut off were generated for different sets of single-several-multimarkers using the GraphPad Prism software version 5. The variable sets evaluated by ROC curves were based on selection of influential variables (VIP > 1.0) from the primary models (Table 1). The cut-off values used for healthy versus diseased was ≥ 10 DF smooth surfaces for baseline caries, and ≥ 18 D smooth surfaces for increment.

## Results

### PLS modelling of a multiplicity of variables against caries in a cross-sectional and prospective setting

A multiplicity of plaque, diet and saliva variables (n = 88) measured at baseline was used to generate prediction (or PLS) models in a cross-sectional (baseline caries) and prospective (2-year caries development) setting in thirty 14-year-old children (Fig. 1). Baseline caries and 2-year caries development (increment, incidence and progression of lesions) were recorded as total numbers of incipient and manifest lesions at all (or defined) tooth surfaces (Fig. 1, Additional file 1). This caries index allowed a wider variation in the dependent caries variable than the traditional DMFs index (Fig. 1B) and, consequently, generated reasonable PLS models with 30 subjects (Table 1) compared to the triplicate of the subjects (n = 90) required to generate equal models based on the DMFS index.

Primary (all variables) and secondary (all influential markers, VIP > 1.0) prediction models were generated by the PLS method (Table 1). Secondary models were also generated from blocks (bacteria - diet - saliva) or sets (single - five - multimarkers) of influential markers for comparative purposes (Table 1).

### Ability of plaque, diet and saliva variables to predict baseline caries and caries development

The primary PLS model from all plaque, diet and saliva variables predicted baseline caries (R^2 ^= 59.7%, Q^2 ^= 26%, model M1), but not caries development (models M2 to M4), at a reasonable level (Table 1). Accordingly, previous caries was required among the baseline variables to predict 2-year caries increment at a reasonable level (R^2 ^= 56.7%, Q^2 ^= 21%, M2). The primary models for incidence (M3) and progressing (M4) of lesions behaved less well.

The secondary models formed from blocks of influential plaque, diet and saliva biomarkers predicted baseline caries in the order of plaque bacteria > diet > saliva (Table 1). Similarly, plaque and diet explained increment somewhat better than incidence while saliva behaved poorly. Only plaque bacteria behaved well as relates to progression.

### Ability of sets of markers to predict baseline caries

**Figure 2:**
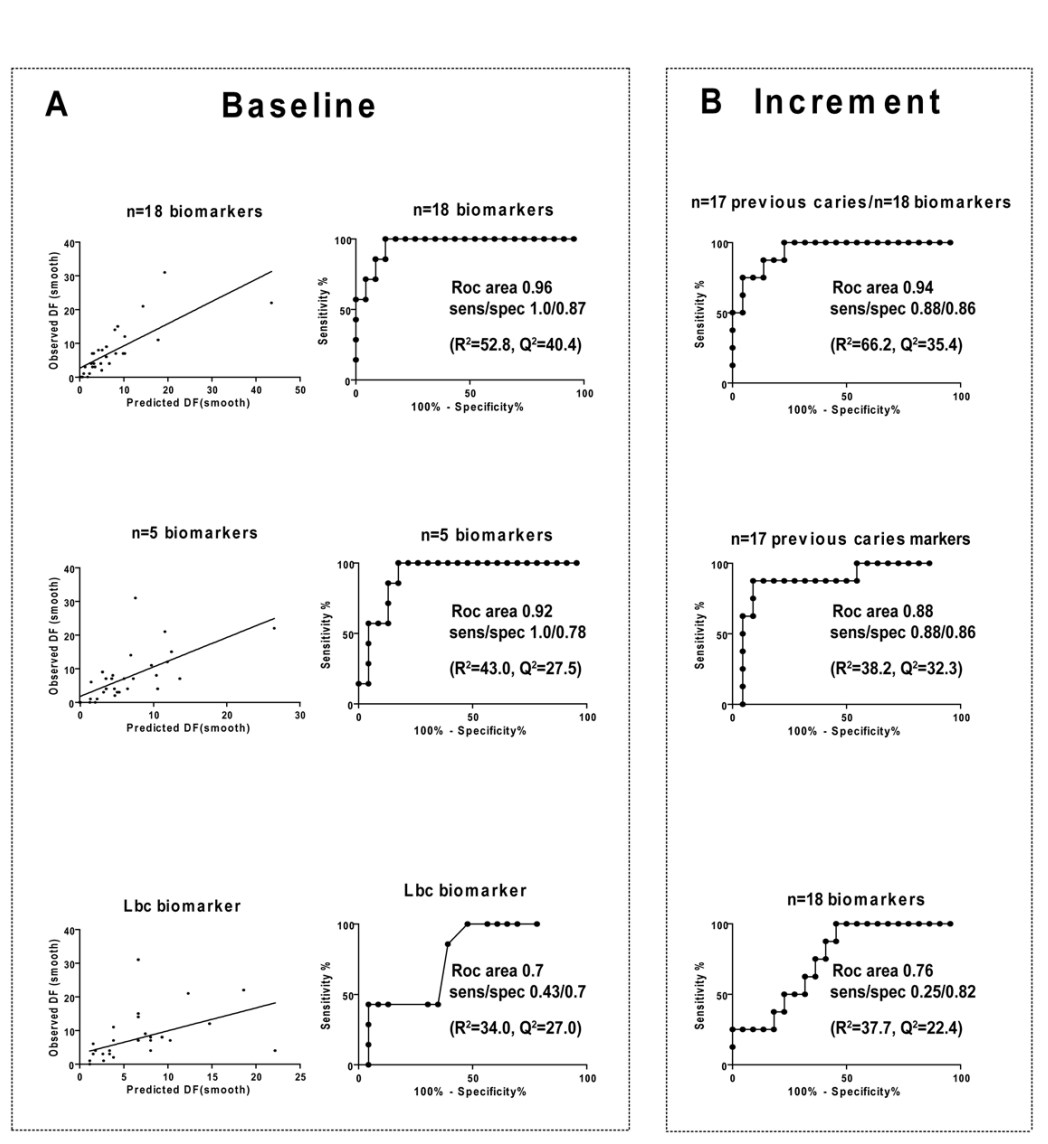
**Ability of multimarkers to predict baseline caries and 2-year increment**. **A**. Ability of influential biological multimarkers (n = 18), a five marker panel (lactobacilli, mutans streptocococci, diet retinol equivalents and zinc and parotid saliva calcium) and a single lactobacilli variable to predict baseline caries as shown by plots of observed versus predicted baseline caries and ROC curves. **B**. Ability of influential biological (n = 18) and previous caries (n = 17) multimarkers alone or together to predict 2-year increment as shown by ROC curves.

We next compared secondary models formed from all influential biomarkers (n = 18 multimarkers), a five biomarker panel and a single lactobacilli marker, for ability to predict baseline caries (Table 1, Fig. 2A). The biomarker sets predicted baseline caries in the order of the n = 18 multimarkers (R^2 ^= 52.8%, Q^2 ^= 40.4%) > five biomarkers (R^2 ^= 43%, Q^2 ^= 27.5%) > single lactobacilli marker (R^2 ^= 34%, Q^2 ^= 27%) (Table 1).

Similarly, observed versus predicted caries and ROC curves displayed a sensitivity and specificity in the order of n = 18 multimarkers (1/0.87, ROC area 0.96) > five markers (1/0.78, ROC area 0.92) > single lactobacilli marker (0.43/0.7, ROC area 0.7), all at 1/3 of the subjects diagnosed sick (Fig. 2A).

### Ability of sets of markers to predict caries increment

We then investigated secondary models formed from all influential biological (n = 18) or previous caries (n = 17) multimarkers alone or combined for ability to predict 2-year caries increment (Table 1, Fig. 2B). The marker sets predicted caries increment in the order of biological/previous caries multimarkers (R^2 ^= 66.2%, Q^2 ^= 35.4%) > previous caries multimarkers alone (R^2 ^= 38.2%, Q^2 ^= 32,3%) > biological multimarkers alone (R^2 ^= 37.7%, Q^2 ^= 22.4%) (Table 1).

Similarly, observed versus predicted caries (data not shown) and ROC curves displayed a sensitivity and specificity in the order of biological/previous caries multimarkers (0.88/0.86 ROC area 0.94) > previous caries multimarkers alone (0.88/0.86, ROC area 0.88) > biological multimarkers alone (0.25/0.82, ROC area 0.76), all at 1/3 of the subjects diagnosed sick (Fig. 2B).

### A wide but shallow gliding scale of biomarkers for caries

**Figure 3:**
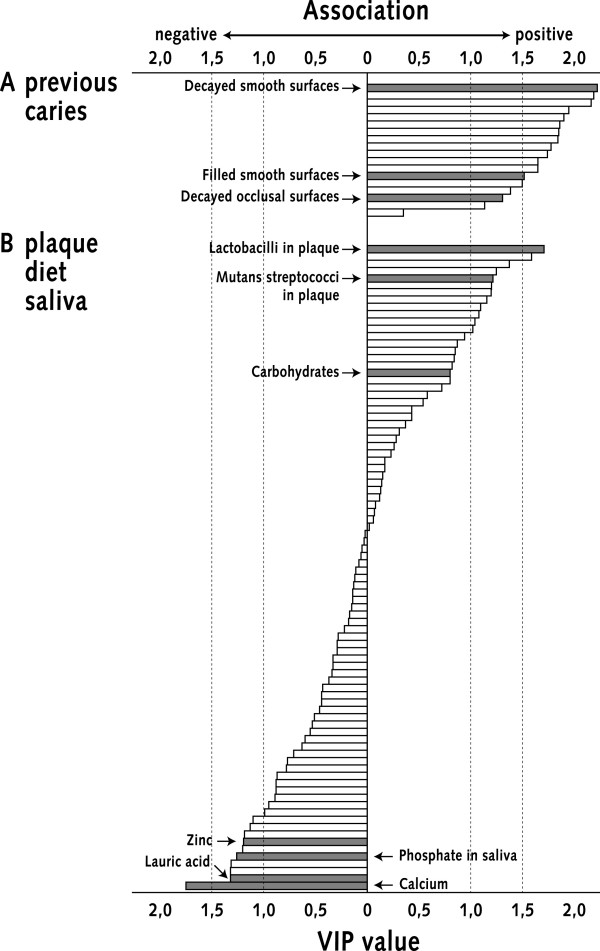
**Wide but shallow gliding scale of variables related to caries**. Schematic picture illustrating the ability of the PLS method to give the relative rank of associations for multiple variables. Displayed is the gliding scale of all plaque, saliva and diet (n = 88) and previous caries (n = 18) variables in the case of incidence (model M4); from the most influential (VIP > 1.0) positively associated variables (top) to the most influential negatively associated variables (bottom). Some variables are marked by arrows and bold bars.

The primary PLS models revealed the association structure and rank for all individual variables as relates to the different caries measures (Table 2, Fig. 3).

About one fifth of the biomarkers displayed influential (VIPs = 1.0-2.5) positive or negative associations with baseline caries or increment/incidence, while the remaining variables had non-influential associations (Table 2, Fig 3). The complex variable patterning for baseline caries was highly reminiscent of those for increment and incidence, while that for progression was less complex with plaque bacteria as the main influential markers (Table 2).

### Potential caries protecting or promoting biomarkers

The biomarker patterning for increment and incidence was as follows (Table 2):

Lactobacilli and mutans streptococci correlated positively with caries regardless of outcome measure. Lactobacilli behaved better than mutans streptococci and their prevalence in plaque better than their counts in saliva. The amount of plaque tended to associate positively, while the opposite was true for fluoride in plaque and rate of plaque formation.

Negative associations occurred for a set of potentially protective diet variables (*e.g*. zinc, lauric acid, calcium etc). Some dietary factors tended to behave in the opposite way (*e.g*. iron, carbohydrates), while sucrose and monosaccharide intake lacked influential (VIPs < 1.0) associations.

The concentration of phosphate in stimulated parotid saliva associated negatively and flow rate positively. Other parotid saliva factors (*e.g*. calcium, S-IgA and saliva aggregation) and whole saliva secretion rate, pH and buffer capacity lacked influential associations.

### Ability of the variable set to predict different types of caries

The primary PLS models also revealed the ability of the variables to predict caries lesions as relates to type (decayed and filled) or localization (affected surfaces). The biological variables predicted baseline caries better for smooth than for occlusal surfaces and decayed surfaces better than filled ones (data not shown). The primary models for increment, incidence and progression behaved in the same way (data not shown), as reflected in the same relative importance of smooth versus occlusal and decayed versus filled surfaces as previous caries markers for caries development (Fig. 3).

## Discussion

The present study indicate the potential value of the PLS method in predicting and understanding the caries disease by showing *i*) an improved ability of multimarkers to predict caries cross-sectional (baseline caries) and prospective (2-year increment) and *ii*) a gliding scale of a multiplicity of known and novel biomarkers for caries. The entire set of plaque, diet and saliva variables (n = 88) displayed a wide but shallow gliding scale of one fifth caries promoting or protecting markers and four fifths non-influential variables (Fig 3). Plaque lactobacilli and mutans streptococci and dietary sugars are such previously known markers and a set of new, potentially caries-protective, diet factors novel ones.

The cross-sectional design generated the strongest models and was therefore used to compare sets (single-five-multimarkers) and blocks (plaque-diet-saliva) of variables. The biological multimarkers (n = 18) explained almost 60% of the variation in baseline caries (R^2 ^= 52.8%, Q^2 ^= 40.4%), and predicted baseline caries well with a sensitivity/specificity of 1/0.87 (ROC area 0.96). While the biological multimarkers (n = 18) predicted caries well, a five marker panel (0.92) predicted less well and a lactobacilli marker (0.7) badly. However, although the R^2 ^values are somewhat better than previously reported for caries, they are weak from a predictive (and chemometric) point of view and the high ROC area values should be treated with caution due to a skewed caries distribution in the sample (Fig. 2). The finding that baseline caries was predicted in the order of a few plaque bacteria > 45 diet variables > a few salivary proteins and electrolytes agrees with lactobacilli and mutans streptococci as markers of an acidified plaque environment, difficulties in recording dietary factors and the need for improved saliva factors in future multimarkers. Accordingly, the redundant character of the present multimarkers should be replaced by improved plaque bacteria and saliva factors in future multimarkers.

The prospective design generated less strong models but evaluated the multimarkers ability to predict caries in a true prediction setting. The biological multimarkers explained 2-year caries development slightly better (R^2 ^= 37.7%, Q^2 ^= 22.4%) than the generally reported 1/3 of the individual variation in caries development explained by biological and previous caries markers together. Moreover, multimarkers from previous caries (ROC area 0.88) alone, or together with biological multimarkers (ROC 0.94), predicted increment with a slightly higher sensitivity/specificity of 0.88/0.86 than reported before. Both the refined caries index recording numbers of incipient and manifest lesions and the multiple caries outcome measures summarizing various etiological factors may account for the improved behaviour of multimarkers also when formed from previous caries.

The present study shows the potential use of PLS to evaluate and generate potential multimarkers for prediction or understanding of caries. For example, the similar marker patterning for baseline caries, increment and incidence but different for progression (with a few and a dominant lactobacilli marker) may, hypothetically, reflect mechanistic differences between initiation and progression of lesions. The PLS technique can be used in small clinical samples and thus simplify measurements of many variables. The present study used 30 subjects and should largely be viewed from a methodological point of view, rather than in terms of individual markers or relevance to the population. We therefore made use of a cohort sampled 1985-87 when caries prediction studies were popular, and for which many plaque, diet and saliva variables already had been recorded. The caries level of the 1985-87 sample is consistent with that period but markedly higher than reported for a corresponding Swedish cohort (n = 3400) ten years later. The present and other sensitive caries indices recording incipient and manifest caries lesions at all surfaces may provide one way to handle today's low level of caries. We do, however, not know the intra-examiner reliability for the index used in this study, but there was only one carefully trained examiner and the intra-examiner reliability is usually high in caries studies. Moreover, although the sensitive index used may overestimate caries, it generated relevant models that behaved similar to those generated by the DMFS index but which required a triplicate of subjects (n = 90) for equally strong models.

The present findings suggest a number of macro (*e.g*. calcium, protein, lauric acid) and micro (*e.g*. zinc, retinol) nutrients with potentially caries protective properties. These nutrients can not be linked to any obvious food item or group, except for calcium which reflects milk and cheese consumption or protein which may reflect a protein-rich diet, why differential eating or behavioural patterns may be at work. Macro and micro nutrients may directly affect oral (host-bacteria) biofilms by modifying salivary pellicle function (as suggested for milk casein peptides [33]), bacterial glycolysis and other enzymatic events (as suggested for medium-chain fatty acids [34] and zinc [35]) or re- and demineralization events (as suggested for calcium and phosphate [36,37]). Macro and micro nutrients like zinc may also indirectly modify the host innate and immune status among children [38,39], and may act in concert to maintain a biofilm homeostasis to prevent dental caries. Finally, the weak positive associations for carbohydrates/sucrose are consistent with the lack of firm links between sugars and caries in many studies.

The poor behaviour of saliva proteins and other quantitative saliva markers, e.g. flow rate, buffer capacity or pH, is striking but consistent with previous notions [40]. The few influential saliva markers, for example calcium and phosphate, are consistent with current re- and demineralization models, but should - as other individual saliva or diet markers - not be over emphasized due to the multiple comparisons. It remains to be determined if saliva key functions and related qualitative protein and allele polymorphisms, such as saliva adhesion of bacteria, PRP and gp-340 protein or peptide polymorphisms, contain more disease-related information than the presently used saliva variables. Finally, screening of saliva by genomic and proteomic approaches could generate markers or multimarker sets for future disease prediction and sub typing of caries based on mechanism(s), another property inherent to the PLS/PCA technology.

## Conclusion

The present study shows the potential of multivariate PLS modelling to handle several-to-multimarker prediction models and suggests a predictive ability in the order of:

i) biological multimarkers > several biomarkers > single biomarkers,

ii) plaque bacteria > diet > saliva biomarkers,

iii) previous caries > biomarkers.

It also shows the potential of PLS modelling to screen and rank a multiplicity of variables for associations with caries in small clinical samples (n = 30): revealing a wide but shallow gliding scale of 1/5th influential caries protecting or promoting and 4/5 non-influential variables.

Biological and previous caries multimarkers behaved somewhat better than previously reported, but future multimarker strategies will require systematic proteomic or genomic searches of improved saliva and plaque bacteria markers.

## Competing interests

The authors declare that they have no competing interests.

## Authors' contributions

ÅN collected the clinical sample (including recorded caries and sampled biological variables), performed PLS analyses and drafted the manuscript. IJ and CK participated in the analyses and drafting of the manuscript. MS designed and supervised the PLS analyses. TE and NS designed and coordinated the study, and NS drafted and designed the final manuscript. All authors read and approved the final manuscript.

## Pre-publication history

The pre-publication history for this paper can be accessed here:

http://www.biomedcentral.com/1472-6831/9/28/prepub

## Supplementary Material

Additional file 1 **description of refined index measuring numbers of incipient and manifest caries lesions at all surfaces**. Click here for file
