# Supplementary material for: Improved ability of biological and previous caries multimarkers to predict caries disease as revealed by multivariate PLS modelling
Source: BMC Oral Health. 2009 Nov 3;9:28. doi: 10.1186/1472-6831-9-28 (PMC2780985; doi:10.1186/1472-6831-9-28)
Supplement: Additional file 1 — description of refined index measuring numbers of incipient and manifest caries lesions at all surfaces. [file 1472-6831-9-28-S1.pdf]

Additional file 1. Refined index for recording of incipient and manifest caries lesions

Tooth condition	Criteria[a] Occlusal surfaces	Buccal and Lingual surfaces	Proximal surfaces	Code
Healthy	Intact fissure. No or very slight discoloration.	Surface intact on probing. No discoloration.	Surface intact on probing. No radiolucence in enamel or dentine.	0
Incipient lesion	Discoloration but no ”catching” on probing.	Narrow white area at the gingiva, smooth on probing.	Radiolucence in the outer half of the enamel.	1
Manifest lesion	Discoloration and/or ”catching” on probing No radiolucence in enamel or dentine.	White area at the gingiva, rough on probing.	Radiolucence in the enamel do not surpass the dentino-enamel junction.	2
”	Cavitation detectable without probing. Radiolucence in the outer half of dentine.	White area at the gingiva with small cavitation on probing.	Radiolucence within the outer half of dentine.	3
”	Cavitation detectable without probing. Radiolucence extending into the inner half of dentine.	Cavitation detectable without. probing.	Radiolucence extending into the inner half of dentine.	4
Filled	Filling.	Filling.	Filling.	5
Recurrent caries	Recurrent caries detectable with or without probing.	Recurrent caries detectable with or without probing.	Recurrent caries detectable with or without probing and/or filling with adjacent radiolucence.	6
Other	Not accessible for registration.	Not accessible for registration.	Not accessible for registration.	7

[a] Decayed (incipient and manifest lesions) and filled surfaces were recorded according to the given criteria. The number of affected surfaces (code 0 to 6) for each subject was utilized in PLS modelling. Fissure sealants were rare but if present they were coded as filled (code 5). No recurrent caries (code 6) was present. Surfaces, either unavailable for scoring due to orthodontic appliances (16 surfaces) or missing due to primary teeth (10 surfaces) were classified as other. No missing permanent teeth occurred. DMFS-values (the D component corresponds to codes 2-4 for occlusal surfaces and 3 and 4 for smooth surfaces) were also calculated.
